# Multicomponent Synthesis of 3,6-Dihydro-2*H*-1,3-thiazine-2-thiones

**DOI:** 10.3390/molecules17021675

**Published:** 2012-02-08

**Authors:** Art Kruithof, Marten L. Ploeger, Elwin Janssen, Madeleine Helliwell, Frans J. J. de Kanter, Eelco Ruijter, Romano V. A. Orru

**Affiliations:** 1 Department of Chemistry and Pharmaceutical Sciences, Amsterdam Institute for Molecules, Medicines and Systems, VU University, De Boelelaan 1083, 1081HV Amsterdam, The Netherlands; 2 School of Chemistry, University of Manchester, Brunswick Street, Manchester, M13 9PL, UK

**Keywords:** 3,6-dihydro-2*H*-1,3-thiazine-2-thiones, cyclic dithiocarbamates, multi-component reactions, molecular complexity, carbon disulfide, 1-azadiene

## Abstract

Non-fused 3,6-dihydro-2*H*-1,3-thiazine-2-thiones constitute a so far rather unexplored class of compounds, with the latest report dating back more than two decades. Thiazine-2-thiones contain an endocyclic dithiocarbamate group, which is often found in pesticides, in substrates for radical chemistry and in synthetic intermediates towards thioureas and amidines. We now report the multicomponent reaction (MCR) of *in situ-*generated 1-azadienes with carbon disulfide. With this reaction, a one-step protocol towards the potentially interesting 3,6-dihydro-2*H*-1,3-thiazine-2-thiones was established and a small library was synthesized.

## 1. Introduction

Multicomponent reactions (MCRs) are very powerful tools in the quest for molecular diversity in the exploration of chemical space [1]. Combining three or more simple starting materials in a one-pot protocol provides quick and easy access to diverse products. In the development of novel MCRs, the use of modular reaction sequences (MRSs) is a useful way of creating diverse scaffolds [1]. MRSs involve the use of an *in situ* generated reactive intermediate, which can be reacted further with a range of different reaction partners, giving rise to a wide variety of scaffolds. An example of the use of MRSs in diversity generating MCRs is the coupling of *in situ* generated 1-azadienes with a range of iso(thio)cyanates [2,3], α-isocyanoesters [4] and amidines [5]. 1-Azadienes thus are versatile intermediates that are able to react as heterodienes, as nucleophiles, or as either 1,2- or 1,4-electrophiles [6].

Based on the analogy of carbon disulfide with other heterocumulenes that react with 1-azadienes, e.g., isothiocyanates, we envisioned the formal hetero Diels-Alder reaction of 1-azadienes with carbon disulfide leading to 3,6-dihydro-2*H*-1,3-thiazine-2-thiones. Using this method, this interesting class of compounds can be synthesized in one step, using only readily available starting materials.

3,6-Dihydro-2*H*-1,3-thiazine-2-thiones contain an endocyclic dithiocarbamate group, which is, e.g., found in protecting groups [7], chiral auxiliaries [8], or in chain transfer agents for RAFT polymerization [9]. Furthermore, (cyclic) organic dithiocarbamates are used as substrates for radical chemistry [10,11,12] and as synthetic intermediates towards thiourea, amidines and guanidines [13,14,15].

The latest report on unfused 3,6-dihydro-2*H*-1,3-thiazine-2-thiones dates back to 1991, when Shutalev *et al*. reported the treatment of α,β-unsaturated ketones with dithiocarbamic acid [16]. Prior to that, only a few reports on the synthesis of 3,6-dihydro-2*H*-1,3-thiazine-2-thiones were published, all using similar procedures [17,18,19,20].

### 2.1. Mechanistic Considerations

**Scheme 1:**
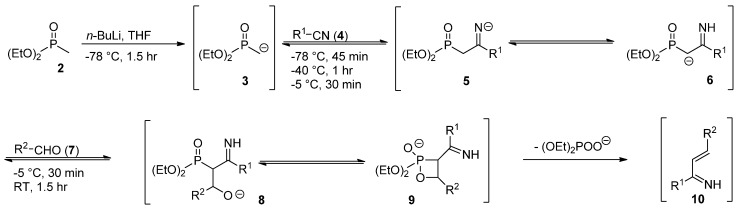
Proposed mechanism for the generation of 1-azadienes **10** [21,22].

The generation of 1-azadienes involves initial nucleophilic attack of the deprotonated diethyl methylphosphonate on the nitrile carbon (Scheme 1) [21,22]. After a proton shift of intermediate **5**, a Horner-Wadsworth-Emmons-type reaction with the aldehyde affords the 1-azadiene **10**.

**Scheme 2:**
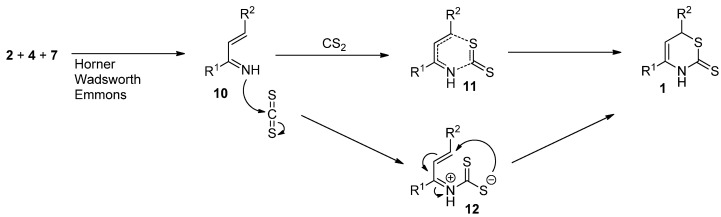
Proposed mechanisms towards 3,6-dihydro-2*H*-1,3-thiazine-2-thiones via *in situ* generated 1-azadienes.

The subsequent reaction with carbon disulfide may proceed by either of two possible routes (Scheme 2). The first possibility is a concerted hetero Diels-Alder-type mechanism, in which the two new bonds (the nitrogen-carbon and the carbon-sulfur bond) are formed simultaneously. The second possibility is a stepwise mechanism, first forming the nitrogen-carbon bond, and forming the carbon-sulfur bond *via* stabilized intermediate **12**.

## 2. Results and Discussion

**Figure 1:**
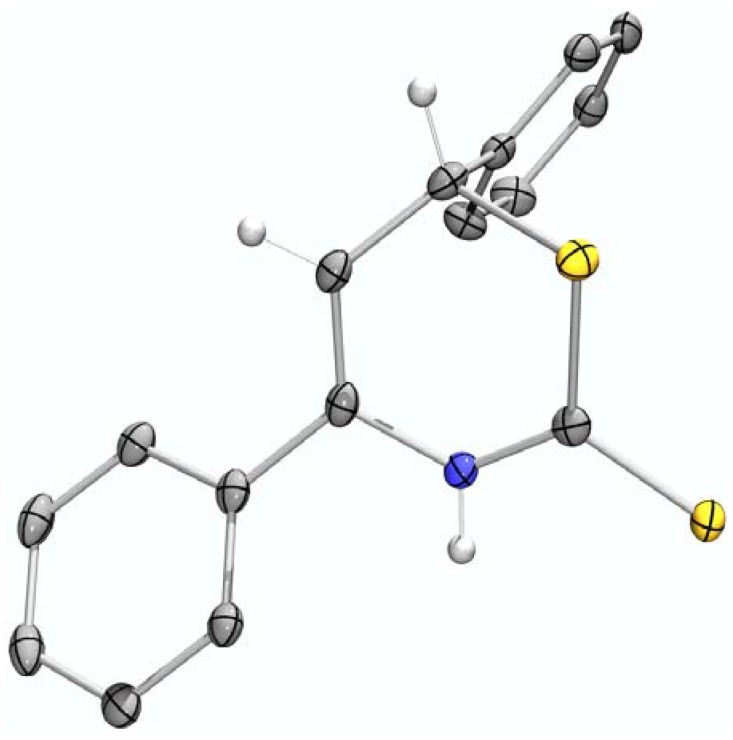
X-ray structure of **1a**.

The 1-azadienes **10** were prepared *in situ* as described by us earlier (Scheme 1) [2]. Carbon disulfide was then added at room temperature. Ten minutes after the addition of carbon disulfide, a quick HPLC analysis of the reaction mixture showed no apparent product formation. Using hexamethylbenzene (C_6_Me_6_) as a quantitative internal standard, crude ^1^H-NMR measurements showed complete product formation after sixteen hours (68%) and only little degradation of the product, even after three days (66%). The structure of 4,6-diphenyl-3,6-dihydro-2*H*-1,3-thiazine-2-thione (**1a**) was confirmed by X-ray crystallography (Figure 1). CCDC 865856 contains the supplementary crystallographic data for this paper. These data can be obtained free of charge via www.ccdc.cam.ac.uk/conts/retrieving.html (or from the CCDC, 12 Union Road, Cambridge CB2 1EZ, UK; fax: +44 1223 336033; e-mail: deposit@ccdc.cam.ac.uk).

### 2.1. Scope

After the optimization of the reaction conditions, a small library of 3,6-dihydro-2*H*-1,3-thiazine-2-thiones was synthesized to study the scope of the reaction. Aliphatic and aromatic, as well as sterically demanding inputs, were used (Table 1).

**Table 1:** The synthesized library of 3,6-dihydro-2*H*-1,3-thiazine-2-thiones.

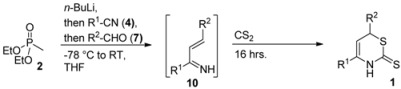				
Entry	R^1^	R^2^	Product	Yield
1	Ph	Ph	**1a**	63% ^[a]^
2	Ph	Ph	**1a**	69% ^[b]^
3	*i*Pr	Ph	**1b**	37% ^[b]^
4	Ph	*i*Pr	**1c**	38% ^[b]^
5	2,6*-*Me_2_C_6_H_3_	2,4,6*-*Me_3_C_6_H_2_	**1d**	0% ^[b]^
6	Ph	2,4,6*-*Me_3_C_6_H_2_	**1e**	30% ^[b]^
7	*p*-F_3_CC_6_H_4_	*o*- F_3_CC_6_H_4_	**1f**	n.d. ^[c]^
8	*p*-F_3_CC_6_H_4_	*p*- MeOC_6_H_4_	**1g**	65% ^[d]^
9	*p*-MeOC_6_H_4_	*o-* F_3_CC_6_H_4_	**1h**	59% ^[a]^
10	*p-*MeOC_6_H_4_	*p*- MeOC_6_H_4_	**1i**	64% ^[b]^
11	*p*- MeOC_6_H_4_	*o*- MeOC_6_H_4_	**1j**	65% ^[a]^
12	*p-*BrC_6_H_4_	*p-*BrC_6_H_4_	**1k**	60% ^[b]^
13	*o*-BrC_6_H_4_	*m-*BrC_6_H_4_	**1l**	72% ^[b]^

^[a]^ Purified by recrystallization from methanol; ^[b]^ Purified by flash column chromatography; ^[c]^ Yield not determined; contains unknown impurities; ^[d]^ Product not fully separated from remaining aldehyde starting material; yield determined by ^1^H-NMR analysis from product/contaminant ratios.

In the formation of the 1-azadienes, 1.2 equiv. of *n*-BuLi were used. In order to prevent reductive dehalogenation in the synthesis of **1k** and **1l** (Table 1, entries 12 and 13, respectively) only 1.05 equivalents of *n*-BuLi were used [23].

Because of the presence of excess of starting materials and side products, attempts were made to purify the crude reaction products by recrystallization. Methanol proved to be a suitable solvent for crystallization of products **1a**, **1h** and **1j**. Only with the addition of water did some of the other entries precipitate, together with starting materials and/or side products. For compounds **1b–e**, **1i**, **1k**, and **1l** purification by crystallization was unsuccessful, and flash chromatographic purification was instead used in those cases. Unfortunately, products **1f** (entry 7) and **1g** (entry 8) could not be isolated in pure form by flash chromatography. In case of **1g** (entry 8), the product could not be separated from the remaining aldehyde starting material, while in case of **1f** (entry 7) the product was contaminated with unknown side products.

Aliphatic nitrile or aldehyde inputs give reduced yields in this reaction (entries 3 and 4), which is in agreement with earlier observations [2,3]. Compound **1d** (entry 5) was not formed, most likely due to steric hindrance, and an equimolar mixture of 2,6-dimethylbenzonitrile and the mesitylene carboxaldehyde was recovered after column chromatography. This suggests that first stage of the reaction, *i.e.*, the nucleophilic attack of the deprotonated phosphonate on the nitrile, did not take place and apparently is particularly sensitive to steric hindrance. The second stage of the reaction, *i.e.*, the Horner-Wadsworth-Emmons-type reaction of the aldehyde, seems less influenced by steric bulk, as indicated by the fact that **1e** (entry 6) is formed, albeit in lower yield (30%). Steric hindrance does not seem to play a significant role in case of mono-*ortho*-substitution (entries 9, 11 and 13), as products **1h**, **1j**, and **1l** are obtained in similar yields as **1a**. Furthermore, electronic factors do not seem to play a significant role, as both electron-donating and electron-withdrawing groups are allowed on both the nitrile and the aldehyde input with comparable yields in all cases (entries 8–13).

Although all products **1** are stable in the solid state, it should be noted that the products slowly decompose over time in solution. This is one of the reasons for the difficulties in purifying some of the products, e.g., **1f** and **1g**. The degradation is more pronounced in crude mixtures in the presence of air, than in purified products kept under inert atmosphere. However, more studies are necessary to pinpoint the nature of this degradation.

## 3. Experimental

### 3.1. General Information

All reactions were carried out under inert atmosphere, unless stated otherwise. ^1^H- and ^13^C- nuclear magnetic resonance (NMR) spectra were recorded on a Bruker Avance 500 (500.23 MHz and 125.78 MHz respectively) or a Bruker Avance 400 (400.13 MHz and 100.61 MHz respectively) instrument with chemical shifts (δ) reported in ppm downfield from tetramethylsilane. Electrospray Ionization (ESI) mass spectrometry was carried out using a Bruker micrOTOF-Q instrument in positive ion mode (capillary potential of 4,500 V). Column chromatography was performed with flash silica gel (40–63 μm) and a mixture of ethyl acetate and cyclohexane. Compounds in Thin Layer Chromatography (TLC) were visualized by UV detection. THF was dried and distilled from sodium potassium alloy and benzophenone prior to use. Diethyl methylphosphonate was prepared as described earlier [3]. Other commercially available reagents were used without further purification. 

### 4.2. General Procedure I for the Formation of 3,6-Dihydro-2*H*-1,3-thiazine-2-thiones

To a solution of diethyl methylphosphonate (292 μL, 2.00 mmol) in dry THF (10 mL, 0.2 M) at −78 °C was added *n*-butyllithium (1.6 M in hexanes), and this mixture was stirred for 1.5 h at this temperature. The nitrile (1.1 equiv.) was added, and stirring was continued at −78 °C for 45 min. Then the reaction mixture was warmed to −40 °C, stirred for 1 h, and subsequently warmed to −5 °C and stirred for 30 min. The aldehyde (1.1 equiv.) was then added, and the reaction mixture was stirred at −5 °C for 30 min, warmed to room temperature, and stirred for an additional 1.5 h. After the addition of carbon disulfide (0.60 mL, 10 mmol) and overnight stirring, the solvent was removed *in vacuo* and either recrystallization from methanol or flash column chromatography afforded the product.

For the reaction time optimization, hexamethylbenzene (50 mg, 0.31 mmol, 0.65 equiv.) was added to the reaction mixture prior to the addition of the carbon disulfide. For each time point, a sample of the reaction mixture was taken, concentrated *in vacuo* and analyzed by ^1^H-NMR.

*4,6-Diphenyl-3,6-dihydro-2*H*-1,3-thiazine-2-thione* (**1a**)


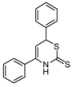
According to General procedure I, the reaction between diethyl methylphosphonate, *n*-butyllithium (1.5 mL, 2.4 mmol, 1.2 equiv), benzonitrile (225 μL, 2.20 mmol), benzaldehyde (224 μL, 2.20 mmol) and carbon disulfide and subsequent flash column chromatography (EtOAc/*c-*hex = 1:15) afforded **1a** as an orange foam (390 mg, 1.38 mmol, 69%). Alternatively, recrystrallization from MeOH afforded **1a** as a light orange solid (356 mg, 1.26 mmol, 63%). ^1^H-NMR (500 MHz, CDCl_3_) δ (ppm) 9.07 (bs, 1H), 7.51–7.43 (m, 5H), 7.41–7.31 (m, 1H), 5.62 (d, *J* = 5 Hz, 1H), 5.09 (d, *J* = 5 Hz, 1H); ^13^C-NMR (126 MHz, CDCl_3_) δ (ppm) 193.5 (C), 139.7 (C), 138.7 (C), 134.6 (C), 130.0 (CH), 129.3 (2 × CH), 129.2 (2 × CH), 128.5 (CH), 128.0 (2 × CH), 126.0 (2 × CH), 103.4 (CH), 46.8 (CH); HRMS [24] [MH^+^] 284.0538 (calc. C_16_H_14_NS_2_, 284.0562) [MH^+^−CS_2_] 208.1108 (calc. C_15_H_14_N, 208.1121); Melting point: 125–128 °C (decomp.).

*4-Isopropyl-6-phenyl-3,6-dihydro-2*H*-1,3-thiazine-2-thione* (**1b**)


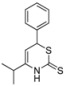
According to General procedure I, the reaction between diethyl methylphosphonate, *n*-butyllithium (1.5 mL, 2.4 mmol, 1.2 equiv.), isobutyronitrile (197 μL, 2.20 mmol), benzaldehyde (224 μL, 2.20 mmol) and carbon disulfide followed by flash column chromatography (EtOAc/*c-*hex = 1:15) afforded **1b** as a yellow solid (185 mg, 0.74 mmol, 37%). ^1^H-NMR (500 MHz, CDCl_3_) δ (ppm) 8.62 (bs, 1H), 7.39–7.24 (m, 5H), 5.15 (d, *J* = 9.5 Hz, 1H), 4.88 (d, *J* = 9.5 Hz, 1H), 2.46 (sept, *J* = 7 Hz, 1H), 1.22 (d, *J* = 7 Hz, 3H), 1.21 (d, *J* = 7Hz, 3H); ^13^C-NMR (126 MHz, CDCl_3_) δ (ppm) 193.3 (C), 143.6 (C), 140.5 (C), 129.1 (2 × CH), 128.3 (CH), 127.8 (2 × CH), 99.6 (CH), 46.3 (CH), 32.4 (CH), 20.8 (CH_3_), 20.7 (CH_3_); HRMS [MH^+^] 250.0712 (calc. C_13_H_16_NS_2_, 250.0719) [MH^+^−CS_2_] 174.1275 (calc. C_12_H_16_N, 174.1277); Melting point: 94–95 °C.

*6-Isopropyl-4-phenyl-3,6-dihydro-2*H*-1,3-thiazine-2-thione* (**1c**)


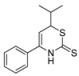
According to General procedure I, the reaction between diethyl methylphosphonate, *n*-butyllithium (1.5 mL, 2.4 mmol, 1.2 equiv.), benzonitrile (225 μL, 2.20 mmol), isobutyraldehyde (200 μL, 2.20 mmol) and carbon disulfide followed by flash column chromatography (EtOAc/*c-*hex = 1:15) afforded 1c as a light yellow solid (192 mg, 0.77 mmol, 38%). ^1^H-NMR (500 MHz, CDCl_3_) δ (ppm) 8.91 (bs, 1H), 7.43 (s, 5H), 5.45 (d, *J* = 5 Hz), 3.68 (t, *J* = 7.5 Hz, 1H), 2.03 (m, 1H), 1.07 (d, *J* = 6.5 Hz, 3H), 1.06 (d, *J* = 6.5 Hz, 3H); ^13^C-NMR (126 MHz, CDCl_3_) δ (ppm) 194.9 (C), 138.4 (C), 134.9 (C), 129.8 (CH), 129.2 (2 × CH), 125.9 (2 × CH), 102.6 (CH), 49.7 (CH), 34.7 (CH), 18.9 (CH_3_), 18.6 (CH_3_); HRMS [MH^+^] 250.0710 (calc. C_13_H_16_NS_2_, 250.0719) [MH^+^−CS_2_] 174.1273 (calc. C_12_H_16_N, 174.1277); Melting point: 93–95 °C.

*6-Mesityl-4-phenyl-3,6-dihydro-2*H*-1,3-thiazine-2-thione* (**1e**)


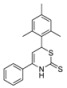
According to General procedure I, the reaction between diethyl methylphosphonate, *n*-butyllithium (1.5 mL, 2.4 mmol, 1.2 equiv.), benzonitrile (225 μL, 2.20 mmol), mesitylene carboxaldehyde (324 μL, 2.20 mmol) and carbon disulfide followed by flash column chromatography (EtOAc/*c-*hex = 1:15) afforded **1e** as a yellow foam (194 mg, 0.60 mmol, 30%). ^1^H-NMR (500 MHz, CDCl_3_) δ (ppm) 9.02 (bs, 1H), 7.48–7.42 (m, 5H), 6.89 (s, 2H), 5.88 (d, *J* = 3.5, 1H), 5.54 (d, *J* = 3.5 Hz, 1H), 2.45 (bs, 6H), 2.28 (s, 3H); ^13^C-NMR (126 MHz, CDCl_3_) δ (ppm) 196.6 (C), 138.3 (3 × C), 137.5 (C), 134.6 (C), 129.9 (C), 129.8 (2 × CH), 129.3 (3 × CH), 125.8 (2 × CH), 105.6 (CH), 42.6 (CH), 20.9 (3 × CH_3_); HRMS [MH^+^] 326.1020 (calc. C_19_H_20_NS_2_, 326.1032) [MH^+^−CS_2_] 250.1585 (calc. C_18_H_20_N, 250.1590).

*6-(2-(Trifluoromethyl)phenyl)-4-(4-(trifluoromethyl)phenyl)-3,6-dihydro-2*H*-1,3-thiazine-2-thione* (**1f**)


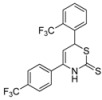
According to General procedure I, the reaction between diethyl methylphosphonate, *n*-butyllithium (1.5 mL, 2.4 mmol, 1.2 equiv.), *p*-trifluoromethylbenzonitrile (376 mg, 2.20 mmol), *o*-trifluoromethylbenzaldehyde (290 μL, 2.20 mmol) and carbon disulfide followed by flash column chromatography (EtOAc/*c-*hex = 1:15) afforded an inseparable mixture of **1f** and impurities (367 mg) ^1^H-NMR (500 MHz, CDCl_3_) δ (ppm) 9.12 (bs, 1H), 8.10–7.20 (m, 8H), 5.60 (d, *J* = 5 Hz, 1H), 5.47 (d, *J* = 5 Hz, 1H); HRMS [MH^+^] 420.0255 (calc. C_18_H_12_F_6_NS_2_, 420.0310) [MH^+^−CS_2_] 344.0847 (calc. C_17_H_12_F_6_N, 344.0868).

*6-(2-methoxyphenyl)-4-(4-(trifluoromethyl)phenyl)-3,6-dihydro-2*H*-1,3-thiazine-2-thione* (**1g**)


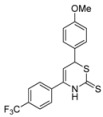
According to General procedure I, the reaction between diethyl methylphosphonate, *n*-butyllithium (1.5 mL, 2.4 mmol, 1.2 equiv.), *n*-butyllithium (1.5 mL, 2.4 mmol, 1.2 equiv.), *p*-trifluoromethylbenzonitrile (376 mg, 2.20 mmol), *p*-methoxybenzaldehyde (335 μL, 2.20 mmol) and carbon disulfide followed by flash column chromatography (EtOAc/*c-*hex = 1:15) afforded an inseparable mixture of **1g** and impurities (599 mg). ^1^H-NMR (500 MHz, CDCl_3_) δ (ppm) 9.10 (bs, 1H), 7.71 (d, *J* = 8 Hz, 2H), 7.61 (d, *J* = 8 Hz, 2H), 7.28 (d, *J* = 9 Hz, 2H), 6.90 (d, *J* = 9 Hz, 2H), 5.67 (d, *J* = 4.5 Hz, 1H), 5.47 (d, *J* = 4.5 Hz, 1H), 3.81 (s, 3H); HRMS [MH^+^] 382.0515 (calc. C_18_H_15_F_3_NOS_2_, 382.0542) [MH^+^−CS_2_] 306.1085 (calc. C_17_H_15_F_3_NO, 306.1100).

*4-(4-Methoxyphenyl)-6-(2-(trifluoromethyl)phenyl)-3,6-dihydro-2*H*-1,3-thiazine-2-thione* (**1h**)


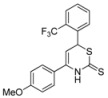
According to General procedure I, the reaction between diethyl methylphosphonate, *n*-butyllithium (1.5 mL, 2.4 mmol, 1.2 equiv.), *p*-methoxybenzonitrile (293 mg, 2.20 mmol), *o*-trifluoromethylbenzaldehyde (290 μL, 2.20 mmol) and carbon disulfide and subsequent recrystallization from MeOH afforded **1h** as a light yellow solid (448 mg, 1.18 mmol, 59%). ^1^H-NMR (500 MHz, CDCl_3_) δ (ppm) 9.09 (bs, 1H), 7.71 (d, *J* = 8 Hz, 1H), 7.67 (d, *J* = 8 Hz, 1H), 7.60 (t, *J* = 7.5 Hz), 7.46-7.38 (m, 3H), 6.95 (d, *J* = 9 Hz, 2H), 5.44 (d, *J* = 5 Hz, 1H), 5.41 (d, *J* = 5 Hz, 1H), 3.84 (s, 3H); ^13^C-NMR (126 MHz, CDCl_3_) δ (ppm) 192.4 (C), 161.0 (C), 139.5 (C), 138.2 (C), 133.0 (CH), 130.8 (CH), 127.4 (q, *J* = 18 Hz, C), 127.4 (2 × CH), 126.0 (q, *J* = 6 Hz, CH), 125.1 (CH) 123.5 (q, *J* = 403 Hz, CF_3_) 114.6 (2 × CH), 101.1 (CH), 55.5 (CH_3_), 42.2 (CH); HRMS [MH^+^] 382.0523 (calc. C_18_H_15_F_3_NOS_2_, 382.0542) [MH^+^−CS_2_] 306.1084 (calc. C_17_H_15_F_3_NO, 306.1100); Melting point: 147–148 °C (decomp.).

*6-(2-Methoxyphenyl)-4-(4-methoxyphenyl)-3,6-dihydro-2*H*-1,3-thiazine-2-thione* (**1i**)


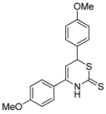
According to General procedure I, the reaction between diethyl methylphosphonate, *n*-butyllithium (1.5 mL, 2.4 mmol, 1.2 equiv.), *p*-methoxybenzonitrile (393 mg, 2.20 mmol), *p*-methoxybenzaldehyde (335 μL, 2.20 mmol) and carbon disulfide followed by flash column chromatography (EtOAc/*c-*hex = 1:15) afforded **1i** as an orange foam (441 mg, 1.28 mmol, 64%). ^1^H-NMR (500 MHz, CDCl_3_) δ (ppm) 9.04 (bs, 1H), 7.40 (d, *J* = 9 Hz, 2H), 7.28 (d, *J* = 9 Hz, 2H), 6.95 (d, *J* = 9 Hz, 2H), 6.89 (d, *J* = 9 Hz, 2H), 5.50 (d, *J* = 5 Hz, 1H), 5.04 (d, *J* = 5 Hz, 1H), 3.84 (s, 3H), 3.80 (s, 3H); ^13^C-NMR (126 MHz, CDCl_3_) δ (ppm) 193.7 (C), 160.9 (C), 159.7 (C), 138.3 (C), 131.7 (C), 129.2 (2 *×* CH), 127.4 (2 *×* CH), 127.0 (C), 114.6 (2 *×* CH), 114.5 (2 *×* CH), 102.5, (CH), 55.5 (CH_3_), 55.4 (CH_3_), 46.4 (CH); HRMS [MH^+^] 344.0741 (calc. C_18_H_18_NO_2_S_2_, 344.0773) [MH^+^−CS_2_] 268.1310 (calc. C_17_H_18_NO_2_, 268.1332).

*6-(2-Methoxyphenyl)-4-(4-methoxyphenyl)-3,6-dihydro-2*H*-1,3-thiazine-2-thione* (**1j**)


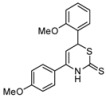
According to General procedure I, the reaction between diethyl methylphosphonate, *n*-butyllithium (1.5 mL, 2.4 mmol, 1.2 equiv.), *p*-methoxybenzonitrile (393 mg, 2.20 mmol), *o*-methoxybenzaldehyde (300 mg, 2.20 mmol) and carbon disulfide and subsequent recrystallization from MeOH afforded **1j** as a light orange solid (448 mg, 1.30 mmol, 65%). ^1^H-NMR (500 MHz, CDCl_3_) δ (ppm) 9.00 (bs, 1H), 7.43 (d, *J* = 8 Hz, 2H, 7.33–7.25 (m, 2H), 6.98–6.93 (m, 3H), 6.90 (d, *J* = 8 Hz, 1H), 5.50 (d, *J* = 6 Hz, 1H), 5.36 (d, *J* = 6 Hz, 1H), 3.87 (s, 3H), 3.85 (s, 3H); ^13^C-NMR (126 MHz, CDCl_3_) δ (ppm) 194.3 (C), 160.8 (C), 156.4 (C), 138.8 (C), 129.4 (CH), 128.2 (CH), 127.4 (2 × CH), 127.2 (C), 120.9 (CH), 114.6 (2 × CH), 110.8 (CH), 100.8 (CH), 55.6 (CH_3_), 55.5 (CH_3_), 40.0 (CH); HRMS [MH^+^] 344.0755 (calc. C_18_H_18_NO_2_S_2_, 344.0773) [MH^+^−CS_2_] 268.1318 (calc. C_17_H_18_NO_2_, 268.1332); Melting point: 139–143 °C (decomp.).

*4,6-Bis(4-bromophenyl)-3,6-dihydro-2*H*-1,3-thiazine-2-thione* (**1k**)


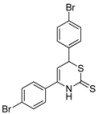
According to General procedure I, the reaction between diethyl methylphosphonate, *n*-butyllithium (1.3 mL, 2.1 mmol, 1.05 equiv.), *p*-bromobenzonitrile (400 mg, 2.20 mmol), *p*-bromobenzaldehyde (407 mg, 2.20 mmol) and carbon disulfide followed by flash column chromatography (EtOAc/*c-*hex = 1:15) afforded **1k** as a red foam (532 mg, 1.21 mmol, 60%). ^1^H-NMR (500 MHz, CDCl_3_) δ (ppm) 9.02 (bs, 1H), 7.58 (d, *J* = 6.5 Hz, 2H), 7.50 (d, *J* = 6.5 Hz, 2H), 7.34 (d, *J* = 6.5 Hz, 2H), 7.22 (d, *J* = 6.5 Hz, 2H), 5.57 (d, *J* = 5 Hz, 1H), 5.01 (d, *J* = 5Hz, 1H); ^13^C-NMR (126 MHz, CDCl_3_) δ (ppm) 193.0 (C), 138.6 (C), 138.1 (C), 133.3 (C), 132.5 (2 × CH), 132.4 (2 × CH), 129.5 (2 × CH), 127.6 (2 × CH), 124.4 (C), 122.7 (C), 103.2 (CH), 46.1 (CH); HRMS [MNa^+^] 461.8557 (calc. C_19_H_11_Br_2_NNaS_2_, 461.8592) [MH^+^−CS_2_] 363.9304 (calc. C_15_H_12_Br_2_N, 363.9331).

*4,6-Bis(4-bromophenyl)-3,6-dihydro-2*H*-1,3-thiazine-2-thione* (**1l**)


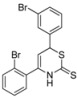
According to General procedure I, the reaction between diethyl methylphosphonate, *n*-butyllithium (1.3 mL, 2.1 mmol, 1.05 eq), *o*-bromobenzonitrile (400 mg, 2.20 mmol), *m*-bromobenzaldehyde (257 μL, 2.20 mmol) and carbon disulfide followed by flash column chromatography (EtOAc/*c-*hex = 1:15) afforded **1l** as a light pink foam (638 mg, 1.45 mmol, 72%). ^1^H-NMR (500 MHz, CDCl_3_) δ (ppm) 8.93 (bs, 1H), 7.66 (d, *J* = 8 Hz, 1H), 7.57 (s, 1H), 7.44 (d, *J* = 7.5, 1H), 7.38 (t, *J* = 3 Hz, 2H), 7.36-7.28 (m, 2H) 7.25 (t, *J* = 6 Hz, 1H), 5.42 (d, *J* = 5 Hz, 1H), 4.97 (d, *J* = 5 Hz, 1H); ^13^C-NMR (126 MHz, CDCl_3_) δ (ppm) 191.9 (C), 142.2 (C), 138.3 (C), 135.3 (C), 133.6 (CH), 131.6 (CH), 131.5 (CH), 131.3 (CH), 131.0 (CH), 130.7 (CH), 128.0 (CH), 126.6 (CH), 123.1 (C), 122.5 (C), 105.1 (CH), 46.1 (CH); HRMS [MNa^+^] 461.8569 (calc. C_19_H_11_Br_2_NNaS_2_, 461.8592) [MH^+^−CS_2_] 363.9309 (calc. C_15_H_12_Br_2_N, 363.9331).

## 4. Conclusions

We report the novel MCR of *in situ* generated 1-azadienes with carbon disulfide to furnish unfused 3,6-dihydro-2*H*-1,3-thiazine-2-thiones in reasonable to good yields. In this reaction, electron-donating as well as electron-withdrawing substituents are tolerated on both the nitrile and aldehyde input. Moreover, aliphatic nitriles and aldehydes can be used in this reaction, although these sometimes lead to lower yields. In conclusion, rapid and easy access to a rather underexplored class of compounds was established with a wide range of potential inputs.

## Supplementary Materials

Supplementary materials can be accessed at: http://www.mdpi.com/1420-3049/17/2/1675/s1.
